# Evaluation of Mandibular Trabecular Microarchitecture in Stafne Bone Cavity: A Pilot Study †

**DOI:** 10.3390/diagnostics16101453

**Published:** 2026-05-10

**Authors:** Nebiha Gozde Ispir, Gokalp Aslan

**Affiliations:** Department of Oral and Dentomaxillofacial Radiology, Faculty of Dentistry, Gazi University, Bişkek Street, 1. Sk. No: 4, Emek, Ankara 06490, Turkey

**Keywords:** Stafne bone cavity, fractal dimension analyses, bone microarchitecture, cone-beam computed tomography, volume dimension

## Abstract

**Background:** We evaluated whether mandibular trabecular microarchitecture, measured via fractal dimension (FD), influences Stafne bone cavity (SBC) development. **Methods:** In this study, 14 images (0.56%) containing SBC were identified among 2500 retrospectively reviewed cone-beam computed tomography (CBCT) images. Among the detected SBCs, those with panoramic radiographs in the archive were included in the study. Two groups were formed based on panoramic images: an SBC group and a control group consisting of individuals without SBC, with a similar sample size. FD analysis was performed using ImageJ version 1.3 software (National Institutes of Health, Bethesda, MD, USA) on the panoramic radiographs of these two groups. Post hoc power analysis and Cohen’s effect size were calculated to evaluate the robustness of the results. **Results:** No significant difference existed between the mean FDs of the SBC and control groups (*p* > 0.05). Mean FD values were slightly lower in SBC regions (1.18 ± 0.22), compared to other mandibular regions (~1.23–1.30). While FD averages were lower within SBC regions, effect size analysis showed negligible differences (d < 0.21) in posterior regions, indicating structural similarity. However, a large effect size (d = −0.85) in the canine–lateral region suggested a localized trend. **Conclusions:** Within the limitations of this pilot study, SBC does not appear to disrupt the surrounding trabecular structure of the mandible. The findings support the static and developmental nature of SBC.

## 1. Introduction

Stafne bone cavity (SBC) is an asymptomatic radiolucent lesion located between the mandibular canal and the base of the mandible in the posterior region. It is usually unilateral, with flat cortical or protruding peripheral surfaces, and can be of different shapes and sizes [1]. SBC has remained one of the most intriguing radiological enigmas in oral and maxillofacial surgery since its first description by Edward Stafne in 1942 [1]. The etiology of these rare radiolucencies is not fully known, and the true nature of its pathogenesis continues to trigger intense academic debate [2]. While major etiological hypotheses gravitate towards the mechanical pressure exerted by submandibular salivary glands or abnormal vascular pulsations of the facial artery, these theories fail to explain why SBC occurs in only a fraction of the population despite the universal presence of these anatomical structures [3,4].

Because SBC is usually asymptomatic, it is diagnosed incidentally during dental radiographic examinations [5]. Diagnosis of SBC is usually made with panoramic radiographs [5]. However, in some cases, it can also be diagnosed using computed tomography (CT), cone-beam computed tomography (CBCT) and magnetic resonance imaging (MRI), which provide more precise images [2].

The structural integrity of the jawbone is determined by its trabecular complexity, which can now be measured using advanced mathematical models. Among these, fractal dimension (FD) analysis is a quantitative method frequently used in many scientific studies to reveal the underlying structure of complex shapes. This quantitative analysis method offers advantages over subjective analyses, as it eliminates operator-dependent differences and yields reproducible results [6]. FD analysis has emerged as a robust, non-invasive computational tool for assessing bone quality and microarchitecture. It can quantitatively provide the trabecular and cortical bone structure of bone tissue (jawbone, maxillary jaw, femur, etc.) [6,7,8].

Although the prevalence and volumetric characteristics of SBCs have been extensively documented in recent years using CBCT [9,10], the underlying “bone quality” of the regions where these cavities form has not yet been investigated. If SBC formation is related to chronic external pressure from adjacent soft tissues, it is reasonable to question whether the mandibular regions in which these defects occur have distinct trabecular microarchitectural characteristics. To our knowledge, no studies have yet integrated 3D volumetric data with 2D fractal microarchitectural analysis to investigate this relationship.

The primary aim of this study was to further explore the relationship between SBC and mandibular trabecular microarchitecture. Accordingly, the null hypothesis was that there would be no difference in mandibular trabecular complexity between individuals with and without SBC. Furthermore, this study aimed to investigate whether the volumetric size of SBC, measured via semi-automatic segmentation on CBCT, correlates with the surrounding bone’s fractal complexity. Accordingly, this research aimed to provide an integrated evaluation of the volumetric and microarchitectural characteristics of SBC. This study was designed as a pilot investigation due to the low prevalence of SBC and the limited number of available cases. A preliminary version of this study was previously presented at the 5th International Congress of the Oral Diagnosis and Maxillofacial Radiology Society [11].

## 2. Methods

In this study, 2500 CBCT images taken of patients aged 18 years and over were retrospectively scanned. Images with SBC were detected in the scanned CBCT. Among the detected SBCs, those with panoramic radiographs in the archive were included in the study. In our study, images obtained using the Planmeca Promax 3D Mid (Planmeca, Helsinki, Finland) CBCT device with imaging areas of 160 × 92 mm and 160 × 52 mm, 0.4 mm voxel size, 90 kVp, 8.0 mA, and a 13.5 s scanning time and images obtained with the Sirona ORTHOPHOS XG (Sirona, Bensheim, Germany) panoramic device with acquisition parameters of 70 kVp, 10 mA, and a 16 s average scanning time were evaluated. CBCT images and panoramic radiography images from patients with a history of trauma or reconstructive surgery, congenital disorders, or artifacts that would affect image quality (metal artifacts, motion artifacts, etc.) were excluded from the study. All panoramic images were obtained using standardized imaging protocols. No additional distortion correction was applied; however, all regions of interest were selected from anatomically consistent areas to minimize the potential influence of image distortion.

In this study, two groups were formed based on panoramic images: an SBC group and a control group consisting of individuals without SBC, with a comparable sample size to the SBC cases identified in the archive. Cases without SBC were selected from individuals with a similar gender distribution and age range to those with SBC. Based on the information available in the archive records regarding systemic diseases and medication use, it was also taken into account that there should be no systemic disease affecting bone structure (hyperparathyroidism, osteoporosis, etc.) or medication use (corticosteroids, bisphosphonates, etc.) in either group.

### 2.1. Determining the Localization of SBCs

The localization of SBCs in panoramic radiographs was evaluated according to tooth alignment and anatomical regions. Accordingly, their localizations were classified as

- right/left mandible first molars alignment,
- right/left mandible second molars alignment,
- right/left mandible canines and lateral incisors alignment,
- right/left angulus mandible region.

### 2.2. Regions of Interest Determination and Fractal Dimension Analysis

**Figure 1:**
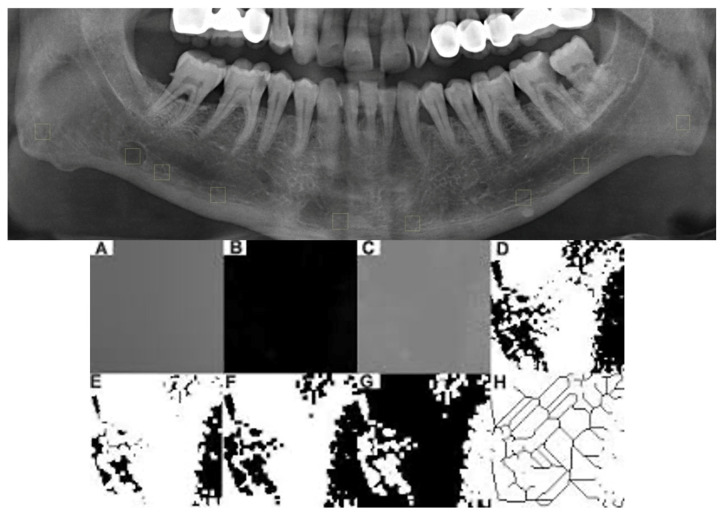
Region of interest (ROI) determination and FD analysis; The highlighted yellow boxes indicate the ROI selected for analysis, (**A**) cropped duplicate, (**B**) Gaussian blur, (**C**) blurred images were subtracted from the original image and 128 gray values were added for each pixel, (**D**) Make Binary, (**E**) Erode, (**F**) Dilate, (**G**) Invert, (**H**) Skeletonize.

FD analysis was performed on panoramic radiographs of individuals with and without SBC, as this method is widely used for evaluating trabecular bone structure in dental imaging. Although CBCT images were used for lesion identification and anatomical assessment, panoramic images were preferred for FD analysis due to the limitations of CBCT in assessing fine trabecular structures, including voxel size, reconstruction parameters and partial volume effects [12]. ImageJ version 1.3 software (National Institutes of Health, Bethesda, MD, USA) was used to perform this analysis. To ensure the standard, considering the previously determined classification of localization of SBC, regions of interest (ROI) were selected bilaterally from four areas in the mandible in each image. These ROI regions were located inferior to the mandibular canal, at the level of the 1st mandibular molar tooth, at the level of the 2nd mandibular molar tooth, at the angulus mandible, and at the level between the canine and lateral teeth. Outside these areas, in images containing SBCs, another ROI was identified at the centers of the relevant SBCs. The ROI size was set at 40 × 40 pixels so that it would not exceed the cortical borders of the smallest-sized SBC detected, would be in the center, and would not include anatomical structures. This size was applied to all images (Figure 1).

In this study, White and Rudolph’s “box counting” method was used [13]. In this method, each of the selected data was cropped and duplicated (Figure 1A). The “Gaussian Blur” filter (σ, 35 pixels) was applied to the duplicated ROI to blur the bright areas resulting from soft tissue and different bone thickness (Figure 1B). The blurred images were then subtracted from the original image. By adding 128 gray values for each pixel, areas of different brightness in the images were distinguished from the trabecular structure of the bone marrow (Figure 1C). In order to distinguish the boundaries of the bone marrow and trabecular structure, the image was converted to a two-color format, black and white, using the “Make Binary” option (Figure 1D). The “Erode” step was applied to reduce noise in the image (Figure 1E). The “Dilate” step was applied to expand and highlight existing areas (Figure 1F). The borders of the trabecular bone were revealed using the “Invert” option by converting the white areas on the image to black and the black areas to white (Figure 1G). Using the “Skeletonize” option, the image showing the trabecular structure was converted to a skeletal structure format (Figure 1H). To calculate the FD value, the image was divided into squares of 2, 3, 4, 6, 8, 12, 16, 32, and 64 pixels using the “Fractal Box Counter” option. Thus, FD values were calculated at different pixel sizes with frames containing trabeculae and the total number of frames in the image.

To assess the reproducibility of manual ROI placement and FD measurements, interobserver reliability was evaluated by two independent examiners who were blinded to each other’s measurements and followed an identical ROI selection protocol (40 × 40 pixels) and image-processing pipeline.

### 2.3. Stafne Bone Cavity Volumetric Dimension Measurement

**Figure 2:**
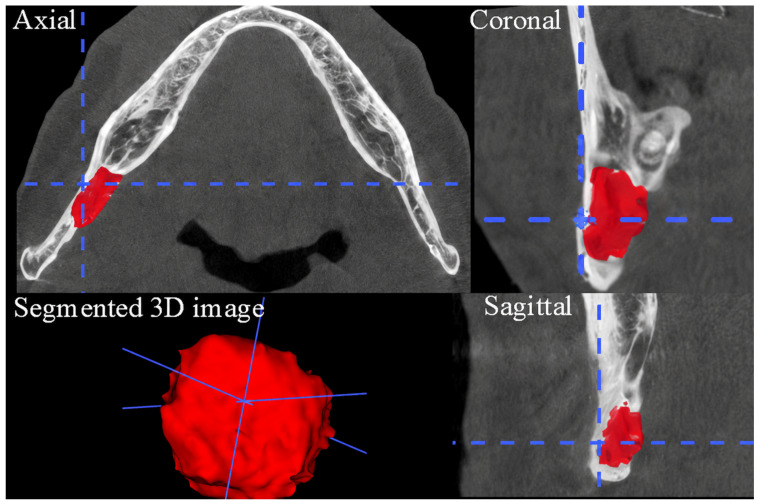
Three-dimensional visualization of SBC and volumetric dimension measurement. Axial, coronal, and sagittal CBCT images demonstrate the anatomical location of the lesion. The segmented area is shown in red, representing the SBC volume obtained through semi-automatic segmentation. Blue dashed lines indicate the crosshair reference axes used for spatial localization of the lesion across orthogonal planes.

CBCT images of individuals with SBC were transferred to the ITK Snap 4.0 (Cognitica, Philadelphia, PA, USA) program in DICOM (digital imaging communications in medicine) format to measure the volumetric size of SBC. The volumetric dimensions of the SBC in mm^3^ were measured using the semi-automatic segmentation feature of the program (0.1 mm slice thickness). In the axial, coronal and sagittal planes, the outermost point of the SBC was selected, and the relevant regions were determined as special areas. Reference points were selected for the relevant SBC. Using the ‘Start segmentation’ command, the program automatically segmented the SBC volume using contrast differences in the grayscale images starting from the selected reference points. Thus, three-dimensional visualization of SBC and volumetric dimension measurement values in mm^3^ were obtained using the relevant software program (Figure 2).

### 2.4. Evaluation of the Anatomical Relationships of SBC

The anatomical relationships of SBCs with surrounding cortical structures were evaluated using CBCT images, based on previously described classification approaches in the literature [9,14]. SBCs were assessed in axial, coronal, and sagittal planes.

Accordingly, the relationship between SBCs and the buccal cortical bone was classified as follows: Type A (no relationship), Type B (contact), Type C (contact with expansion), and Type D (cortical perforation).

For the mandibular inferior cortex, SBCs were categorized as follows: Type A (no relationship), Type B (contact), or Type C (complete involvement with cortical perforation).

For the mandibular canal, the relationship was classified as follows: no relationship, contact, or the mandibular canal passing through the SBC.

In the present study, only the categories observed within the dataset were included in the statistical analysis due to the limited sample size.

### 2.5. Statistical Analysis

Data analyses were performed using Statistical Package for Social Science (IBM SPSS) v23 (Inc., Chicago, IL, USA) for Windows software. Normal distribution was examined using the Shapiro–Wilk Test. To compare quantitative data with normal distribution in two-category independent variables, an independent two-sample *t*-test was used to analyze the data. The comparison of quantitative data that did not show a normal distribution in two-category independent variables was analyzed using the Mann–Whitney U test. The Kruskal–Wallis H test was used to compare quantitative variables that did not show normal distribution in three or more groups. The relationship between quantitative variables that did not show normal distribution was examined using Spearman’s rho correlation. Descriptive statistics of quantitative variables were given as mean ± standard deviation and median (minimum–maximum). Intraclass correlation coefficients (ICC) were calculated using a two-way random-effects model with absolute agreement (ICC [2,1]). The significance level was set at *p* < 0.05.

### 2.6. Statistical Power and Effect Size Analysis

**Figure 3:**
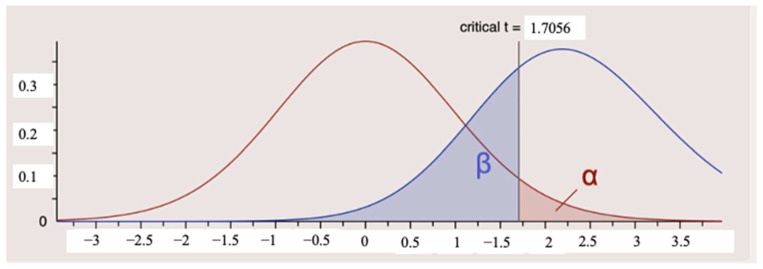
Post hoc power analysis plot generated via G*Power. The red curve represents the null distribution (H_0), and the blue curve represents the actual distribution (H_1) based on the observed large effect size (d = 0.85). With a critical t value of 1.7056 and α = 0.05, the achieved statistical power (1 − β) is 0.71.

Due to the low clinical prevalence of SBC, the sample size was determined by using a systematic screening of 2500 consecutive images. To evaluate the reliability of our findings, a post hoc power analysis was conducted using G*Power software (v3.1.9.6). For the canine–lateral region, where the *p*-value was borderline (0.067), the statistical power (1 − β) was calculated as 0.71, based on a large effect size (Cohen’s d = −0.85). For the posterior regions, effect sizes were small (d < 0.21), indicating minimal magnitude of difference between groups. However, given the limited sample size, these findings should be interpreted cautiously. The present study should therefore be considered exploratory and hypothesis-generating rather than confirmatory (Figure 3).

## 3. Results

In this study, 14 patients (0.56%) with SBC were identified from 2500 retrospectively scanned CBCT images. The mean age of patients with SBC was 59.57 ± 12.68 (min 43—max 83); 21.4% were female and 78.6% were male. The mean age of the control group, which was created by randomly selecting 14 patient images without SBC from other scanned CBCT images, was 65.79 ± 11.79 (min 44–max 82) and consisted of 30% female and 70% male individuals.

All SBCs were localized in the mandibular second molar region, on either the right or the left side. Therefore, subgroup comparison of FD values according to SBC localization could not be performed.

Interobserver reliability analysis demonstrated excellent agreement across all mandibular regions. ICC values ranged between 0.91 and 0.96, indicating high reproducibility of manual ROI placement and FD calculation. All ICC estimates were statistically significant (*p* < 0.001). These findings suggest that minor variability in ROI positioning is unlikely to have materially influenced FD measurements.

**Figure 4:**
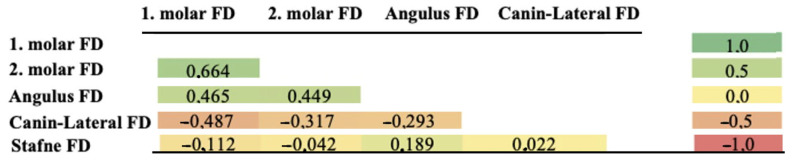
Heat map showing the relationship between regions in those with SBC.

No statistically significant relationship was found between FD values within the SBC region and other regions in individuals with SBC (*p* > 0.05). However, the FD averages of the SBC region were lower than those of other regions (Table 1, Figure 4).

There was no statistically significant difference between the mean FDs of the regions in those with and without SBC (*p* > 0.05) (Table 2). The largest effect size was observed in the canine–lateral region (d = −0.85), although this difference did not reach statistical significance.

The mean SBC volume was found to be 635.6 ± 460.8 mm^3^ (minimum 90 mm^3^, maximum 1567 mm^3^). When the relationship between SBC volume and FD was examined, there was a weak positive correlation. However, this relationship was not statistically significant (*p* > 0.05) (Table 3).

No statistically significant differences were found in FD values according to the anatomical relationships of SBC with the surrounding trabecular structure (*p* > 0.05) (Table 4). When analyzed separately, no significant differences were observed with respect to the buccal cortical bone (*p* = 0.230), mandibular inferior cortex (*p* = 0.116), or mandibular canal (*p* = 0.638).

## 4. Discussion

SBCs are rare asymptomatic lesions. In the present study, the prevalence of SBC was found to be 0.56%. There are many studies in the literature evaluating the prevalence of SBC [9,10,15,16,17,18]. In studies using panoramic radiography, the prevalence of SBC was reported to be from 0.08% to 0.125% [9,15,16]. In studies conducted using CBCT images, this rate was reported to be between 0.48% and 3.5% [9,10]. However, a recent systematic review, including cases diagnosed using CBCT, panoramic, magnetic resonance imaging, and computed tomography, highlighted that the prevalence of SBC fluctuates between 0.03% and 3.55%. [17]. A meta-analysis study evaluating the prevalence of SBC reported this rate as 0.17%. [18]. The results of our study are consistent with the general ranges found in previous studies.

In the present study, the number of male patients with SBC was approximately four times higher than that of female patients, and the mean age of patients with SBC was 59.57 ± 12.68. Previous studies have proven that SBC is more common in males than in females [18]. Additionally, it has been stated in many studies that the average age at which SBC is seen is between 50 and 60 years old [9,17]. Our results are consistent with previous studies that evaluated SBC in terms of age and gender.

Although the etiology of SBC is not fully known, different opinions have been presented in the literature [18]. The opinion that failure of bone accumulation in an area previously containing cartilage led to the development of the defect was first proposed by Stafne [19]. The first surgical examination of the SBC was made by Peterson in 1944 and supported Stafne’s view that it may be of embryonic origin [20]. In 1946, Rushton defended the view that SBC was actually a bone cyst [21]. Early studies suggested a congenital or embryological origin [22]. However, subsequent reports describing cases occurring in middle age have challenged this hypothesis [23]. Histopathological examinations have revealed the presence of tissues associated with the salivary gland; therefore, it has been suggested that the etiology of SBC is related to the involvement of the salivary gland in the mandible during the ossification process [24]. In addition, various findings such as lymphoid tissue, muscle, fat, pleomorphic adenoma of the submandibular gland, and blood vessels have been identified in past studies [4,25]. Recent studies emphasize that no single etiological theory fully explains its occurrence, suggesting that multiple anatomical and physiological factors may contribute to its development [17,18]. In our study, the trabecular structure of the mandibular bone was examined with FD to contribute to the literature regarding etiological factors.

The usefulness of FD analysis in medicine and dentistry has been well established in the context of human biology and bone microarchitecture [6]. Among the available methods for describing trabecular architecture, FD analysis provides a quantitative and reproducible approach for estimating bone quality on radiographic images [26]. Therefore, in the present study, FD was used to evaluate the trabecular structure of the mandibular bone. To the best of our knowledge, no previous studies have specifically investigated the relationship between SBC and mandibular trabecular microarchitecture using FD analysis, limiting direct comparison with the existing literature. In the present study, FD values within the SBC region were numerically lower than those observed in other mandibular regions. Lower FD values are generally associated with reduced structural complexity and may reflect decreased bone density or simplified trabecular patterns [7]. This observation may suggest that regions with relatively lower trabecular complexity could be more susceptible to the development of SBC, although no causal relationship can be inferred from the present data. In addition, although no statistically significant differences were identified between groups, the relatively large effect size observed in the canine–lateral region (d = −0.85) may indicate a potential localized variation in trabecular complexity. This finding should be interpreted with caution due to the limited sample size and lack of statistical significance; however, it may indicate a potential region-specific variation that warrants further investigation. Further studies with larger sample sizes are required to clarify this observation and its potential clinical relevance.

In this study, no statistically significant difference was found between the mean FD regions of those with and without SBC, leading to the conclusion that there was no difference in trabecular bone structure between these two groups. Our primary null hypothesis questioned whether the regions where SBCs form are inherently more ‘structurally complex’ or ‘simpler’ in terms of trabecular complexity. The results do not support this hypothesis. The lack of significant difference in FD values between the SBC and control groups suggests that the underlying bone quality is preserved despite the presence of the SBC. The trabecular microarchitecture in regions associated with SBC appears to remain comparable to that of healthy controls, indicating preservation of structural complexity. These findings may be compatible with a non-aggressive structural presentation of SBC. The preservation of trabecular complexity supports the view that SBC does not represent an intrinsic pathological process affecting bone microarchitecture but may instead reflect an adaptive response to adjacent soft tissue structures. An aggressive bone-destructive process would typically be expected to alter trabecular complexity; however, such alteration was not observed in the present study. Despite rare reports of progressive growth or pathological entities such as pleomorphic adenoma within SBC [4], the absence of significant differences in FD values compared to healthy bone in the present study may be consistent with the concept that SBC represents a relatively static anatomical entity. This may suggest that even in the presence of extrinsic pressure or glandular tissue, the fundamental complexity of the mandibular trabecular bone does not appear to be substantially altered.

For the secondary purpose of this study, the volume of the SBC was calculated. Volumetric measurement in CBCT images can be performed using various software programs. The main ones among these programs are ITK-SNAP (ITK-SNAP 2.4.0; Cognitica, Philadelphia, PA, USA), Mimics (Materialise HQ, Leuven, Belgium), Dolphin 3D (Dolphin Imaging and Management Solutions, Chatsworth, Los Angeles, CA, USA) [27]. These programs enable volumetric measurements by segmenting the examined structures and their boundaries [27]. Previous studies have shown that different software programs are reliable for volume measurement of anatomical structures such as the upper respiratory tract, sphenoid sinus, nasal cavity, and mandibular condyle volume [28,29,30,31,32]. We used ITK-SNAP in the present study because this software program is free and has an easy application and accessibility. There are very few studies in the literature that evaluate the volumetric dimension of SBC [9,33,34,35]. Adisen et al. stated that they found the average volume of the SBC to be 361.7 mm^3^ (minimum 160 mm^3^, maximum 520 mm^3^) [34]. In a study by Koc et al., the average volume of the SBC was found to be 338.05 mm^3^ (minimum 6.14 mm^3^, maximum 1001.32 mm^3^) [33]. In another study by Bagcı and Peker, the volume was stated to be 416.18 ± 180.88 mm^3^ (the smallest volume is 197.4 mm^3^, the largest volume is 859.6 mm^3^) [9]. Sawhney et al. found the volume of SBC to be 332.5 mm^3^ (the smallest volume was 146 mm^3^, the largest volume was 650 mm^3^) in their six-month follow-up study and reported that there was no statistically significant difference in the volumes of the SBCs after six months [35]. In this study, the SBC volume was 635.6 mm^3^ (the smallest volume was 90 mm^3^, and the largest was 1567 mm^3^). Previous studies and the present study results suggest that SBC volumes can vary. These variations may be due to the evaluation of different populations. This study also evaluated the relationship between the FD of SBC and the volume of SBC. However, no statistically significant relationship was found between volume and FD. A comparison could not be made because such research has not been conducted before. Furthermore, the absence of a significant correlation between SBC volume and FD indicates that SBC size does not appear to be directly associated with trabecular complexity. Some authors have proposed that somatic enlargement of the submandibular or sublingual gland, along with local pressure applied to the mandible, may contribute to SBC formation [18].

In the present pilot study, subgroup analyses based on the anatomical relationships of SBC with surrounding cortical structures did not reveal any statistically significant differences in FD values. These findings may suggest that variations in the spatial relationship of SBC with adjacent cortical boundaries, including the buccal cortex, mandibular inferior cortex, and mandibular canal, do not appear to substantially influence trabecular microarchitecture. Within the limitations of the present study, this observation may be consistent with the concept that SBC represents a non-aggressive and relatively static anatomical entity rather than a process associated with localized bone remodeling or structural alteration. Even in cases where SBC demonstrates close proximity or contact with critical anatomical structures, the underlying trabecular complexity appears to remain largely preserved. From a clinical perspective, these preliminary findings may indicate that differences in the anatomical configuration of SBC are unlikely to necessitate different diagnostic or management approaches; however, these interpretations should be made with caution and confirmed by studies with larger sample sizes. Despite its innovative approach, this study also has some limitations. The most important limitation of this study is the low sample size. However, when looking at previous studies, the prevalence of SBC is found to be low [17,18]. This limitation may actually highlight the fact that SBC is a rare lesion and that its etiology is not fully known. Another limitation is that the study is retrospective and does not include surgical procedures or biopsies. SBC is a lesion that generally does not require treatment but should be monitored [36]. Therefore, considering ethical values, the inclusion of individuals in surgical procedures was out of the question for this study. All images were obtained from a single archive, which may limit the generalizability of the findings across different ethnic populations or age groups. The other limitation of the present study is the use of 2D panoramic radiographs for FD analysis, despite the integration of 3D CBCT data for the initial SBC identification. One important methodological limitation relates to the use of two-dimensional FD analysis instead of three-dimensional CBCT-based trabecular evaluation. Although CBCT provides volumetric data, its relatively large voxel size limits the accurate visualization of fine trabecular structures due to partial volume effects, which may affect the reliability of 3D microarchitectural assessment [12]. Previous studies have also reported that FD measurements can be influenced by imaging modality and voxel resolution [12]. Therefore, 2D FD analysis, despite its limitations, remains a widely accepted and reproducible method for evaluating trabecular complexity in dental radiology. While 2D projections may not fully capture the entire volumetric complexity of the 3D trabecular network, 2D FD analysis is a widely validated and robust method for bone quality assessment in dental research. In particular, previous literature has highlighted that the large voxel size required for imaging in CBCT results in inadequate visualization of fine trabeculae and that FD can be strongly influenced by voxel size [12]. Therefore, the 2D FD approach used here remains a reliable tool for measuring mandibular structural stability in SBC cases.

Given the limited sample size and retrospective design, the present findings should be considered exploratory. Within these constraints, no measurable alteration in mandibular trabecular microarchitecture was identified in association with SBC. Although FD values within the SBC region were numerically lower than in other mandibular areas, these differences did not reach statistical significance when compared with healthy controls. Furthermore, no significant correlation was observed between SBC volume and FD, indicating that SBC size does not appear to be directly associated with trabecular complexity.

Taken together, these results are compatible with the concept that SBC represents a structural variation rather than a bone-destructive pathological entity. Larger multicenter investigations integrating advanced three-dimensional microarchitectural analyses are warranted to further clarify the structural characteristics of SBC.

## 5. Conclusions

SBC is a rare mandibular entity with variable volumetric presentations. In the present study, FD values within the SBC region were numerically lower than those observed in other mandibular regions. However, no significant differences were identified between individuals with and without SBC in terms of trabecular microarchitecture. Within the limitations of this pilot study, these findings may suggest that SBC does not appear to be associated with substantial alterations in mandibular trabecular structure and may be more consistent with a developmental variation rather than an aggressive bone-destructive process. Further evaluation of mandibular bone structure in relation to adjacent soft tissues and other anatomical factors may provide additional insights into the etiology of SBC. Future studies using larger sample sizes and multidisciplinary approaches are needed to confirm and expand upon these preliminary findings.

## Figures and Tables

**Table 1:** Examining the relationship between FD values according to region in patients with SBC.

	Mean ± S. Deviation	Median (Min–Max)	Stafne FD	
			r	*p*
1. molar FD	1.23 ± 0.11	1.25 (0.97–1.36)	−0.112	0.703 ^x^
2. molar FD	1.26 ± 0.12	1.28 (0.89–1.38)	−0.042	0.887 ^x^
angulus FD	1.29 ± 0.08	1.29 (1.13–1.4)	0.189	0.517 ^x^
canine–lateral FD	1.25 ± 0.03	1.26 (1.18–1.3)	0.022	0.940 ^x^
Stafne FD	1.18 ± 0.22	1.27 (0.72–1.41)		

^x^ Spearman’s rho correlation.

**Table 2:** Comparison of FDs of regions with and without SBC.

	Stafne Bone Cavity			*p*	Effect Size (d)
	Presence	Absence	Total		
1. molar FD	1.25 ± 0.07	1.23 ± 0.11	1.24 ± 0.09	0.854	0.18
2. molar FD	1.25 ± 0.09	1.26 ± 0.12	1.25 ± 0.1	0.427	−0.09
angulus FD	1.3 ± 0.08	1.29 ± 0.08	1.3 ± 0.08	0.511	0.12
canine–lateral FD	1.22 ± 0.04	1.25 ± 0.03	1.23 ± 0.04	0.067	−0.85

Data are presented as mean ± standard deviation. For independent samples, a *t*-test or Mann–Whitney U test was used as appropriate. Effect sizes are reported as Cohen’s d.

**Table 3:** Correlation between SBC volume and FD.

		Stafne Volume
Stafne FD	r	0.348
	*p*	0.223
	*n*	14

Spearman’s rho correlations.

**Table 4:** Comparison of fractal dimension (FD) values according to anatomical relationships of SBC.

	Groups	Mean ± SD	*p*-Value
Buccal cortical bone	Type A	1.16 ± 0.20	0.230
	Type B	1.21 ± 0.28	
Mandibular inferior cortex	Type A	1.10 ± 0.27	0.116
	Type B	1.28 ± 0.05	
Mandibular canal	Not related	1.26 ± 0.04	0.638
	Related	1.09 ± 0.29	
	Passing through	1.23 ± 0.19	

The Mann–Whitney U test, independent samples *t*-test, and Kruskal–Wallis test were used as appropriate.

## Data Availability

The original contributions presented in this study are included in the article. Further inquiries can be directed to the corresponding author.

## Abbreviations

SBC	Stafne bone cavity
FD	fractal dimension
CBCT	cone-beam computed tomography
Min	minimum
Max	maximum
mm^3^	cubic millimeters
S. Deviation	standard deviation
